# Organ toxicity attenuation by nanomicelles containing curcuminoids: Comparing the protective effects on tissues oxidative damage induced by diazinon

**DOI:** 10.22038/ijbms.2018.23229.5874

**Published:** 2019-01

**Authors:** Mohammadreza Abdollahzadeh Estakhri, Mohammad Shokrzadeh, Mahmoud Reza Jaafari, Mohammad Karami, Hamidreza Mohammadi

**Affiliations:** 1 Pharmaceutical Sciences Research Center, Faculty of Pharmacy and Student Research Committee, Mazandaran University of Medical Sciences, Sari, Iran; 2 Department of Toxicology and Pharmacology, Faculty of Pharmacy, Mazandaran University of Medical Sciences, Sari, Iran; 3 Pharmacutical Science Research Center, Mazandaran University of Medical Sciences, Sari, Iran; 4 Nanotechnology Research Center, Pharmaceutical Technology Institute, Mashhad University of Medical Sciences, Mashhad, Iran

**Keywords:** Antioxidant, Curcumin, Diazinon, Nanocurcumin, Oxidative stress

## Abstract

**Objective(s):**

Diazinon (DZ) is an organophosphate pesticide that induces oxidative damage in different organs. The aim of this study was to compare the effectiveness of nanomicelles containing curcuminoids (NCUR) and natural curcumin (CUR) in attenuating the oxidative damage induced by DZ in male rats.

**Materials and Methods:**

After a single intraperitoneal (IP) injection of DZ (100 mg/kg), the rats were administered either CUR or NCUR (25 and 60 mg/kg, IP). Biomarkers of cell damage including, alanine transaminase (ALT), aspartate transaminase (AST), alkaline phosphatase (ALP), creatinine (Cr), urea, lactate dehydrogenase (LDH), creatine kinase-MB isoenzyme (CK-MB) and troponin I, were quantified in the serum. Lipid peroxidation (LPO) and glutathione (GSH) content in the liver, kidney, and heart tissues were determined.

**Results:**

DZ administration increased the serum levels of ALT, AST, ALP, Cr, urea, LDH, CK-MB, and troponin I; however, the levels significantly (*P*<0.001) decreased in the CUR- and NCUR-treated groups compared to those in the DZ group. NCUR significantly decreased LPO (*P*<0.05) and increased GSH (*P*<0.05) in the heart, kidney, and liver tissues at all doses (especially, at 60 mg/kg) compared with CUR

**Conclusion:**

Our findings suggest that NCUR treatment counters DZ-induced oxidative tissue damage to a greater extent than CUR.

## Introduction

Diazinon (DZ) is a highly toxic organophosphate (OP) pesticide that oxidatively bioactivates to diazoxon by the liver microsomal enzymes system. Inhibition of the cholinesterase enzymes is the major action of DZ that leads to high concentration levels of acetylcholine in the synaptic cleft that causes hypercholinergic symptoms (1, 2). It has been reported that OPs have negative effects on different tissues and organs such as the liver, cardiac, kidney, pancreas, immune system, reproductive system, and vascular walls and can induce liver toxicity, neurotoxicity, cardiotoxicity, genotoxicity or cytotoxicity, and apoptosis. Various biochemical and hematological adverse changes in the body can be induced by OPs compounds (3-7). Moreover, different studies have shown that DZ could induce oxidative damage by increasing the formation of reactive oxygen species (ROS), depletion of the antioxidant enzymes, mitochondrial membrane damage, protein and lipid peroxidation (LPO) and DNA fragmentation in the cells (4, 6, 8-10). DZ is a lipophilic compound with a long half-life and this characteristic property of DZ can cause more circulation in the body, which creates more adverse pathological effects in different tissues (11).

Medicinal plants, as potential sources of health-promoting compounds, have been used to treat various human diseases for thousands of years. Curcuma has been used traditionally in medicine and has a wide spectrum of biological and pharmacological activities. Curcumin (CUR), the active ingredient of Curcuma, is a hydrophobic bioactive yellow polyphenolic compound isolated from the rhizome of the *Curcuma longa* L. (Zingiberaceae) plant. CUR (C_21_H_20_O_6_) with the chemical name of (1E, 6E)-1, 7-bis (4-hydroxy- 3-methoxyphenyl) -1, 6- heptadiene-3, 5-dione, is a bright yellow-orange powder with a melting point of 183 °C. CUR belongs to the class of curcuminoids and is very similar to diarylheptanoids. In the CUR structure, two aromatic ring systems (phenols) are connected by two α, β-unsaturated carbonyl groups. Commercial CUR contains about 17% demethoxycurcumin, 77% diferuloylmethane and 6% bisdemethoxycurcumin. CUR has multiple activities such as ROS inhibitory, anti-inflammatory, antiapoptosis, antibacterial, antiatherogenic, anticancer, and immunomodulatory effects, which were reported in different studies (12-14). Usage of Curcuma, as a herbal medicine, has a long history for the treatment of numerous diseases such as atherosclerosis, diabetes, cancers, digestive disorders, and infectious, liver, and rheumatoid diseases (15-17). In spite of these advantages of CUR, rapid metabolism, rapid systemic elimination, low aqueous solubility, low gastrointestinal absorption, and alkaline pH degradation are the major causes of the decrease in bioavailability and limitation of the clinical usage of the CUR (18).

Nanotechnology is a growing scientific field and considered to be the technology of the future. Over the last decade, various emphases have been given to improve the biodistribution of natural CUR, but recently, nanotechnology has considerably improved the therapeutic effects of CUR. Different nanoparticles such as polymeric nanoparticles, liposomes, niosomes, micelles, nanogels, dendrimers, cyclodextrins, silvers, and solid lipids are emerging as the valuable alternatives to deliver therapeutic applications of CUR (19, 20). The use of nanoparticles for drug delivery purposes appeared to provide CUR with improved permeability, longer circulation, and stronger resistance to metabolic processes (19, 21).

It was stated that CUR can reduce DZ-induced toxicity but low bioavailability is the major limitation of its application in OPs poisoning (22). Hence, use of nanomicelles containing CUR (NCUR) is a novel drug delivery model because of its high bioavailability in aqueous solutions, controlled drug release property, and higher physical stability and drug loading (23). 

In this study, nanocurcumin was prepared as nanomicelles in a series of novel nano-microparticulate systems to improve its aqueous solubility and stability, which was published as our previous patent (23). The present study was designed to evaluate the protective effects of synthesized NCUR against DZ-induced organ toxicity through different methods such as biochemical biomarkers (alanine transaminase (ALT), aspartate transaminase (AST), alkaline phosphatase (ALP), creatinine (Cr), urea, lactate dehydrogenase (LDH), creatine kinase-MB isoenzyme (CK-MB), and troponin I) evaluation in rat treated sera. Furthermore, the efficacy of NCUR on lipid peroxidation (LPO) and glutathione content (GSH) in rat liver, kidney, and heart tissues were investigated after administration of DZ and all experiments were compared with natural CUR. 

## Materials and Methods

***Chemicals***

Diazinon (O, O-diethyl-o-[2-isopropyl-6-methyl-4-pyrimidinyl]-phosphorothioate, 97% purity) was prepared from Shimi Keshavarz CO, (Tehran, Iran). Malondialdehyde (MDA); phosphate-buffered saline (PBS), thiobarbituric acid (TBA), DTNB [5, 5’ dithiobis-(2-nitrobenzoic acid)], and CUR (1E, 6E),7-bis (4-hydroxy- 3-methoxyphenyl) -1,6- heptadiene-3,5-dione), were purchased from Sigma-Aldrich (Steinheim, Germany). Nanomicelles containing curcumin (NCUR) were synthesized and characterized as described earlier (Iranian patent no. 83515, dated July 8, 2014) using GRAS excipients and was used in this study. The Troponin I enzyme-linked immunosorbent assay (ELISA) kit was purchased from Koma biotech Inc, Korea. Alanine transaminase (ALT), aspartate transaminase (AST), alkaline phosphatase (ALP), lactate dehydrogenase (LDH), and creatine kinase-MB isoenzyme (CK-MB) kits were purchased from Biorexfars, UK.

**Figure 1:**
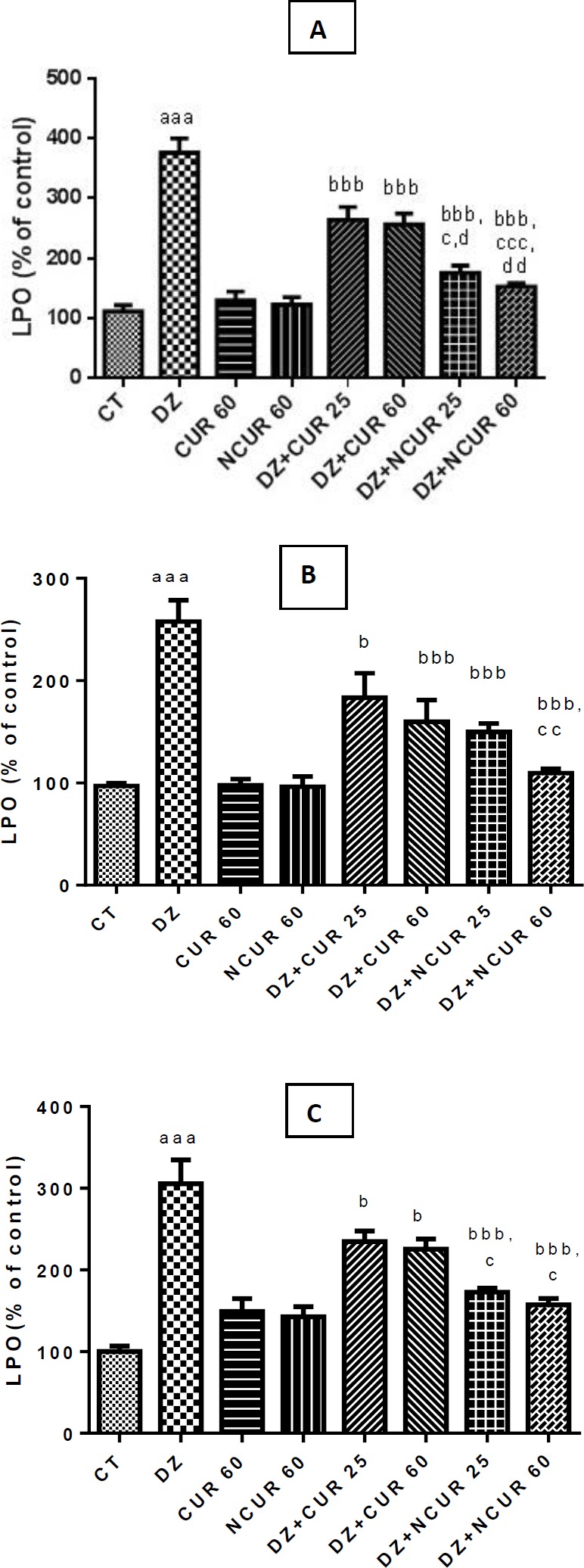
Effects of curcumin (CUR) and nanomicelles containing curcumin (NCUR) treatment on lipid peroxidation in rat liver (A), heart (B) and kidney (C) tissues after administration of Diazinon (DZ). Data are shown as mean±SD (n=6)

^aaa^ *P*<0.001 , ^aa^*P*<0.01 and ^a^*P*<0.05 compared with the control group (CT)

^bbb^ *P*<0.001, ^bb^*P*<0.01, ^b^*P*<0.05 compared with the DZ group

^ccc^ *P*<0.001, ^cc^*P*<0.01, ^c^*P*<0.05 compared with the DZ+CUR (25 mg/kg) group

^dd^ *P*<0.01 and ^d^*P*<0.05 compared with the DZ+CUR (60 mg/kg) group

**Figure 2:**
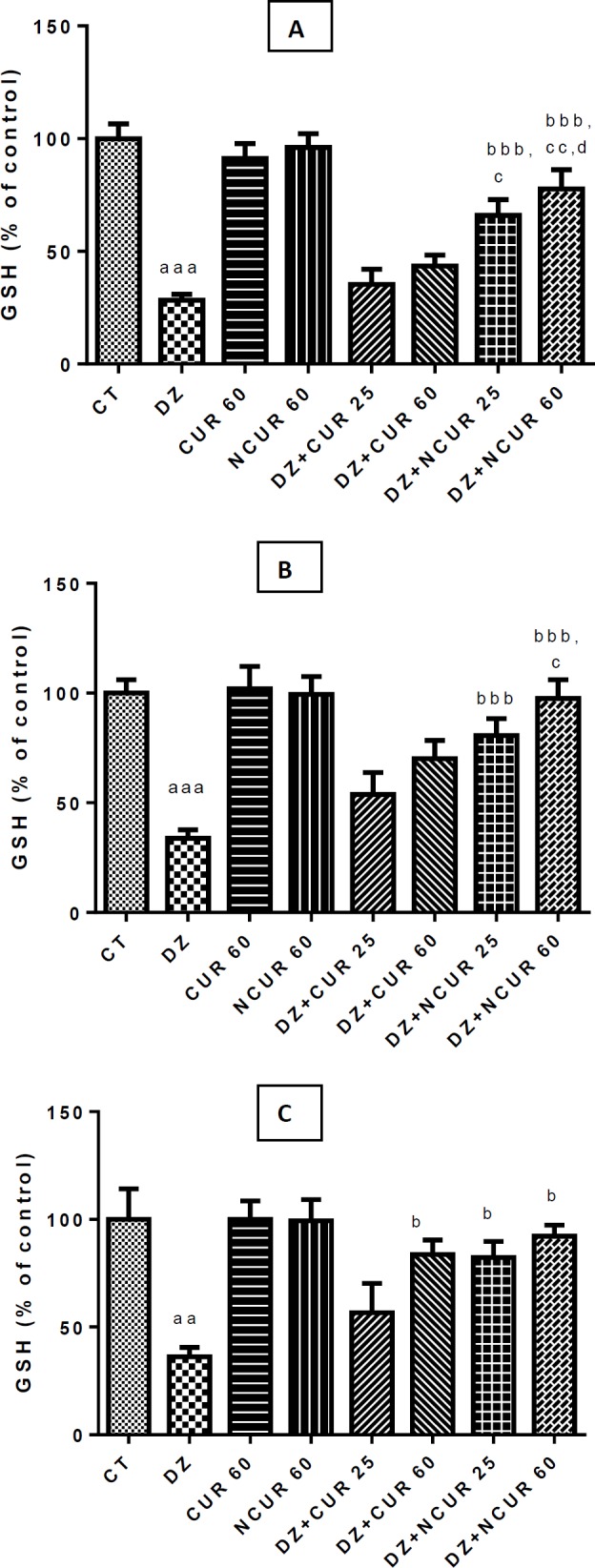
Effects of curcumin (CUR) and nanomicelles containing curcumin (NCUR) treatment on GSH in rat heart (A) kidney (B) and liver (C) tissues after administration of Diazinon (DZ). Data are shown as mean±SD (n=6)

^aaa^ *P*<0.001 and ^aa ^*P*<0.01 compared with the control group (CT)

^bbb^ *P*<0.001, ^bb ^*P*<0.01, ^b ^*P*<0.05 compared with the DZ group

^cc^ *P*<0.01, ^c ^*P*<0.05 compared with the DZ+CUR (25 mg/kg) group

^d^ *P*<0.05 compared with the DZ+CUR (60 mg/kg) group

**Table 1:** Effects of curcumin (CUR) and nanocurcumin (NCUR) treatment on biochemical parameters after induced toxicity by Diazinon (DZ) in rats. Data are presented as percentage of changes to the control group. Data are shown as mean ± SD (n=6).

**Parameter**	**Control**	**DZ**	**CUR60**	**NCUR60**	**DZ+CUR25**	**DZ+CUR60**	**DZ+NCUR25**	**DZ+NCUR60**
**Urea(mg/dl)**	**100±7.92**	**140.2± 9.70** aaa	**94.43± 8.46**	**88.56± 11.05**	**126.7±18.30**	**116.1± 16.57** b	**103.8± 5.90**	**91.06±5.39** cc
**Creatinin(mg/dl)**	**100± 22.61**	**317.6± 55.72** aa	**205.4± 30.82**	**173± 62.22**	**193.5±30.55** b	**194.6± 51.99** b	**162.2±42.73** cc	**104.9±16.70** ccc
**ALT (U/L)**	**100 ± 15.24**	**185.8 ± 18.29** aaa	**101.3 ± 18.47**	**96.76±12.61**	**166±11.13**	**149.8± 14.68**	**128.8 ±7.01**	**118.4±8.20** ccc **,** dd
**AST (U/L)**	**100.0 ±8.31**	**165.7 ±9.14** aaa	**96.95±6.74** bbb	**97.48±6.93** bbb	**119.9±5.36** bb	**130.2±10.60** bbb	**100.3±4.04** ccc **,** d	**94.17±3.32** ccc **,** dd
**ALP (U/L)**	**100.0± 8.28**	**149.0±7.36** aa	**119.4±11.80**	**113.4±12.22**	**118.7±6.31**	**119.4±13.55**	**91.72±5.58** cc	**97.46±9.47** cc
**LDH (U/L)**	**100.0±10.31**	**194.1±30.59** aaa	**105.4±9.37**	**109.1 ±3.82**	**163.7±23.07**	**99.15±8.42** bb	**127.1±21.99** c	**83.48±7.79** ccc **,** ee
**CKMB(U/L)**	**100.0±19.62**	**297.7±63.10** aa	**117.0±14.87**	**98.97± 16.56**	**241.8±55.49**	**213.7±33.73** b	**168.8±14.49** cc	**142.8±18.10** cc **,** dd
**TroponinI(ng/ ml)**	**100.0 ±56.00**	**1359±344.1** aa	**164.4± 57.82**	**183.0± 50.07**	**1130±127.2**	**1079±127.0**	**988.0±135.7**	**621.9±150.9** cc **,d**

aaa P<0.001 ,

aa P<0.01 and

a p<0.05 compared with the control group.

bbb P<0.001,

bb P<0.01,

b P<0.05 compared with the DZ group.

ccc P<0.001 ,

cc P<0.01,

c p<0.05 compared with the CUR (25 mg/kg) group.

dd P<0.01,

d p<0.05 compared with the CUR (60 mg/kg) group.

ee P<0.01,

e p<0.05 compared with the NCUR(25 mg/kg) group.

***Preparation of nanomicelles containing curcumin (NCUR)***

Nanomicelle containing CUR is a registered CUR product (SinaCurcumin®) which has been developed in the Nanotechnology Research Center of Mashhad University of Medical Science and marketed by Exir Nano Sina Company in Tehran-Iran (IRC:1228225765). Each soft gel of SinaCurcumin® contains 80 mg of CUR in the form of nanomicelles (24). These nanomicelles are prepared from GRAS (generally recognized as safe) pharmaceutical excipients and C3-complex (Bisdemethoxycurcumin (2.2% to 6.5%), demethoxycurcumin (15% to 19%), and CUR (75% to 81%)). For preparation of the NCUR, the obtained CUR solved with carrier polysorbate 80, Soy oil, and alpha-tocopherol then, solved acid ascorbic in water was added to it. The morphological feature of NCUR was determined using transmission electron microscopy (TEM). CUR in this structure was located inside the micelles and the results of the particle size analysis with DLS showed that the average particle size was about 10 nm; the polydispersity index (PDI < 0.2), indicating the narrow size distribution of the samples.

***Animal study***

Male Wistar rats (weight 150–200 g) were obtained from the animal house of the Faculty of Pharmacy at Mazandaran University of Medical Sciences (MAZUMS) and were placed on a 12-hr light/dark cycle, at a temperature of 22 ^o^C with free access to the water and standard laboratory diet. All experimental protocols were approved by the Ethical Committee of MAZUMS (ethics number: IR.MAZUMS.REC.92.1671). In this study, 48 adult male rats were divided into 8 groups (n= 6) as follows:

 Group1: control group (received corn oil: normal saline 5:95, v/v).

 Group 2; Only DZ (DZ dissolved in minimum amount of corn oil and reached 0.5 ml with normal saline and administered IP at sublethal dose; 100 mg/kg).

Group 3: (CUR, 60 mg/kg, dissolved in a minimum amount of corn oil and reached 0.5 ml with normal saline and administered IP).

Group 4: (NCUR, 60 mg/kg)

Group 5: DZ (100 mg/kg) + CUR (25 mg/kg) 

Group 6: DZ (100 mg/kg) + CUR (60 mg/kg) 

Group 7: DZ (100 mg/kg) + NCUR (25 mg/kg)

Group 8 : DZ (100 mg/kg) + NCUR (60 mg/kg)

 The DZ solution (100 mg/ml) was made freshly and after 15 min of induced acute toxicity by DZ, treatments with CUR and NCUR at different doses were started. DZ, CUR, and NCUR were injected (single dose, IP). The animal study protocol and selected doses were determined based on previous studies (25-29).

***Blood and tissue sampling***

After 24 hr of the last dose of administration, animals were euthanized by ether and their blood was obtained through cardiac puncture. Serum was removed and used for biochemical experiments. Liver, kidney, and heart tissues were dissected quickly and washed with cold saline and were homogenized (1:10 w/v) in phosphate-buffered saline (PBS) (50 mM sodium phosphate buffer, pH=7.4). Homogenated tissues were centrifuged at 10000 g for 15 min at 4 ^°^C, and supernatants were used for determination of lipid peroxidation (LPO) and glutathione (GSH) content. 

***Biochemical assays and analysis***

Enzyme activities of ALT, AST, ALP, LDH, CK-MB, Troponin I, urea, and creatinine were assessed using kits and were expressed as international units per liter (IU/L).

***Determination of lipid peroxidation (LPO) in tissues***

Malondialdehyde (MDA), as the main marker of lipid peroxidation, was measured in the heart, kidney, and liver tissues. Levels of MDA were measured according to the Fernandez *et al.* method (30) using the spectrophotometric measurement of color developed by reaction of MDA with thiobarbituric acid (TBA). Briefly, phosphoric acid (3 ml, 1%) and TBA (1 ml, 0.6%) were added to 0.5 ml of each sample in a falcon tube and the mixture was incubated for 45 min in a boiling water bath. Then the mixture was cooled and n-butanol (4 ml) was added to the mixture and after that, it was vortexed (1 min) and then centrifuged at 3000 g for 20 min. The created organic layer was separated and its absorbance was calculated at 532 nm using a spectrometer (UV-1601 PC, Shimadzu, Japan).

***Determination of glutathione (GSH) content in tissues***

Glutathione (GSH) content was evaluated in heart, kidney and liver tissues by the Moron *et al.* method (31). The basis of the work was the formation of the yellow color after adding DTNB [5, 5’ dithiobis-(2-nitrobenzoic acid)] to compounds containing sulfhydryl groups. For this purpose, 300 µl of homogenates were blended with 300 µl of 10% tricolor acetic acid (TCA) and vortexed. After centrifugation at 2500 g for 10 min, the upper layers were removed and mixed with the reaction mixture containing 2 ml phosphate buffer (pH: 8) and then 500 µl of DTNB (4%) reagent was prepared and mixed with citrate sodium 10%. After 10 min, the absorbance was evaluated at 412 nm using a spectrophotometer (UV-1601 PC, Shimadzu, Japan). In the end, the amount of GSH was determined based on a standard curve drawn with commercially available GSH. The GSH levels were expressed as nmol/mg protein.

***Statistical analysis***

All values were expressed as mean±SD of several experiments. Differences between the groups were determined using one-way analysis of variance (ANOVA) and Tukey’s as *post hoc* testing was performed for intergroup comparisons using the Graph Pad Prism5 software. Values were regarded as significantly different at *P*<0.05.

## Results

***Effects of CUR and NCUR on serum biochemical parameters in DZ-treated rats***

For determination of liver, kidney, and heart damage and evaluation of the protective effects of CUR and NCUR against DZ-induced damage, the activity of the hepatic enzymes (ALT, AST, ALP, and LDH), kidney biomarkers (urea, Cr), and cardiac damage biomarkers (CK-MB, troponin I) were investigated. Following 24 hr after DZ administration, changes of several assessed parameters indicated the occurrence of liver, kidney, and heart injuries (Table 1). As seen in Table 1, the levels of serum ALT, AST, ALP, LDH, Cr, urea, CK-MB, and troponin I were significantly increased in the DZ group compared to the control (CT) group. Nevertheless, these tissue damage biomarkers were recovered partially by treatment of CUR and/or completely by NCUR (Table 1). The results of this study showed that urea and Cr, as biomarkers of kidney damage, were increased significantly (*P*<0.001) against DZ-induced kidney toxicity. The CUR (60 mg/kg), DZ+NCUR (25 mg/kg), and DZ+NCUR (60 mg/kg) groups could significantly improve these biomarkers when compared with the DZ group. In comparison with DZ+CUR (25 mg/kg) and DZ+CUR (60 mg/kg) groups, DZ+NCUR (60 mg/kg) showed significant effects on decreasing of raised urea and Cr by DZ (Table 1). 

In this study, the levels of ALT, AST, ALP, and LDH, as indicators of liver toxicity, significantly increased in the DZ group compared to the control group (*P*<0.001) (Table 1). A significant (*P*<0.001) decrease of AST and ALP was observed in the DZ+NCUR (25 mg/kg) group compared with DZ+CUR (25 mg/kg) and DZ+CUR (60 mg/kg) treated groups. Also, significant (*P*<0.01, *P*<0.001) decrease of AST and ALP in DZ+NCUR (60 mg/kg) group was observed when compared with DZ+CUR (25 mg/kg) and DZ+CUR (60 mg/kg) groups. ALT was significantly decreased in the DZ+NCUR (25 mg/kg) group in comparison with the DZ+CUR (25 mg/kg) group. ALT also significantly decreased in the DZ+NCUR (60 mg/kg) group when compared with DZ+CUR (25 mg/kg) and DZ +CUR (60 mg/kg) (*P*<0.001, *P*<0.05) groups, respectively. LDH significantly reduced in DZ+NCUR (25 mg/kg) and DZ+NCUR (60 mg/kg) groups in comparison with the DZ +CUR (25 mg/kg) group (Table 1). 

Our results showed that CK-MB and troponin I as heart damage factors were increased significantly by DZ administration. CK-MB was decreased significantly (*P*<0.001) in DZ+NCUR (25 mg/kg), DZ+NCUR (60 mg/kg), and DZ+CUR (60 mg/kg) (*P*<0.05) groups in comparison with the DZ group. Moreover, significant differences between the DZ+NCUR (25 mg/kg) group in comparison with the DZ+CUR (25 mg/kg) group, and the DZ+NCUR (60 mg/kg) group compared with the DZ+CUR (25 mg/kg) group were observed that imply the significant effectiveness of NCUR. Also, DZ+NCUR (60 mg/kg) could significantly (*P*<0.05) decline the elevated troponin I when compared with DZ+CUR (25 mg/kg) and DZ+CUR (60 mg/kg) (Table 1).

***Effects of CUR and NCUR on the oxidative stress biomarkers and antioxidant status in DZ- treated rats***

The results of the present study indicated that MDA levels, as lipid peroxidation indicator, significantly increased (*P*<0.001) in liver, heart and kidney tissues after administration of DZ when compared with the CT group (Figures 1A, B, C); whereas LPO levels were decreased significantly in CUR and NCUR groups at all doses after administration of DZ in comparison with the DZ group. Moreover, NCUR at 60 mg/kg, could significantly decrease MDA levels in all of the tested tissues compared to different doses of CUR (Figures 1A, B, C). A significant decrease of LPO was observed by DZ+NCUR (25 and 60 mg/kg) when compared to DZ+CUR (25 and 60 mg/kg) in liver tissues treated with DZ (Figure 1A). 

DZ treatment caused a significant decline (*P*<0.001) of GSH content in the heart, liver and kidney tissues compared to the control group (Figures 2A, B, C). However, the content of glutathione significantly (*P*<0.001) increased in the heart only by NCUR at all doses (25 and 60 mg/kg). No significant change of GSH amount was observed in heart tissue of rats that received only CUR at all doses compared to the DZ group (Figure 2A). Moreover, NCUR but not CUR, could significantly (*P*<0.001) increase GSH levels in kidney tissues at different doses (25 and 60 mg/kg) after administration of DZ (Figure 2B). GSH content in the liver showed a significant (*P*<0.001) rise in DZ+NCUR (25 and 60 mg/kg) and only DZ+CUR (60 mg/kg) groups when compared with the DZ group (Figure 2C). However, 60 mg/kg dose of NCUR showed more significant effects on attenuation of oxidative damage induced by DZ in all experiments (Figures 1, 2A, B, C). 

## Discussion

Results of the present study indicated that single-dose treatment with NCUR attenuates DZ-induced oxidative damage in the liver, heart, and kidney tissues of rats. We found that NCUR has better antioxidant efficacy than CUR.

DZ, a commonly used organophosphate pesticide, is a potent cholinesterase inhibitor. Organophosphates cause hemostatic disorders and metabolic disturbances (1). They have been found to cause oxidative stress by increasing the level of reactive oxygen species (ROS) in several organs after acute and chronic exposure (4, 6). 

Biomarkers of oxidative tissue damage include ALT, AST, ALP, LDH, urea, Cr, CK-MB, and troponin I; we found that DZ increases the levels of these biomarkers in the heart, liver, and kidney tissues. Treatment with both CUR and NCUR significantly attenuated these levels. Moreover, the antioxidant effect of NCUR was significantly greater than that of CUR (Table 1). 

CUR is a potent scavenger of small intracellular oxidative molecules. It scavenges free-radicals by easily transferring electrons or donating H atoms from its two phenolic groups (32). Moreover, it up-regulates the expression of *O*-6-methylguanine-DNA methyltransferase, a DNA repair gene, and improves tissue antioxidant status (33). Furthermore, CUR increases heme oxygenase activity and inhibits the function of inducible nitric oxide synthase (13, 34). These natural antioxidant properties of CUR can be attributed to its hydrogen-donating phenolic groups or the chain-breaking property of its molecular structure. Previous reports have shown that CUR exhibits anticancer and antioxidant properties by regulating the expression of genes that are necessary for the functioning of activator protein 1 and nuclear factor κβ (17, 35).

Despite these advantages, CUR is poorly bioavailable due to its rapid metabolism, low aqueous solubility, low gastrointestinal absorption, and degradation at alkaline pH; therefore, it has limited clinical utility (18). To address this issue, different nanoparticulate formulations, such as polymeric, silver, and solid lipid nanoparticles, as well as liposomes, niosomes, micelles, nanogels, dendrimers, and cyclodextrins are being evaluated (19, 36, 37). 

 Several CUR nanoparticles have been reported to show positive and protective effects on different organs (19, 38). They are also reported to provide targeted protection against oxidative stress-induced cell death through various mechanisms (37, 39). Despite numerous reports on the beneficial effects of CUR on DZ-induced toxicity (22, 40), the utility of a nanoformulation of CUR in preventing organophosphate-induced toxicity in comparison to that of CUR remains unexplored. Hence, we formulated nanomicelles of CUR (23) for evaluation of its beneficial effects on DZ-induced oxidative tissue damage and compared its effectiveness with that of free CUR. 

Single-dose administration of DZ (100 mg/kg, IP) significantly increased the serum ALT, AST, ALP, Cr, urea, CK-MB, and troponin I, indicating oxidative damage to the liver, kidney, and heart tissues. In support of our study findings, it was reported that DZ increases the level of LPO in the liver (41), heart, and kidney (8, 10) due to ROS generation.

Damage to hepatocytes is reflected by an increase in liver-specific enzymes, such as ALT, AST, LDH, and ALP, which are released into the blood circulation after cellular damage (42). Our results were in accordance with previous findings of DZ-induced oxidative liver damage (3, 4, 10, 42-44). We observed that treatment with CUR and NCUR significantly decreased these liver-specific enzymes. We also showed that NCUR exhibited better liver-protective effects than free CUR.

DZ is reported to increase blood urea and Cr levels, and cause histological changes in the kidney (8). We observed that elevated serum urea levels were significantly reduced by treatment with both NCUR (at all doses) and CUR (only at 60 mg/kg); Cr levels were decreased by NCUR and CUR at all doses. The significant reduction in urea and Cr levels, observed with NCUR (60 mg/kg) treatment compared to that of CUR treatment after DZ insult, implies better effectiveness of NCUR than CUR.

Elevated serum LDH indicates myocardial infarction and cellular death; dying cells release this isoenzyme into the circulation (45). Therefore, increased serum LDH and CK-MB, observed in our study, confirms that DZ damages the membrane integrity of myocardial cells. Cardiotoxicity is reported to be accompanied with increased serum LDH, CK-MB, and troponin I levels in DZ-exposed rats (46, 47). Accordingly, we observed that DZ increased the levels of these biomarkers of cardiotoxicity; however, NCUR at doses of 25 and 60 mg/kg attenuated these levels. Further, it was more effective than CUR in this context (Table 1). 

Results of the current study showed that DZ significantly increased the LPO level in the liver, kidney, and heart tissues; however, both CUR and NCUR reduced the level of LPO (Figure 1A, B, C). This suggests that CUR and NCUR attenuate DZ-induced toxicity in the liver, kidney, and heart. These results are consistent with earlier reports of the efficacy of CUR in preventing DZ toxicity (22, 40). However, we demonstrated that the synthesized NCUR offers better protection than CUR against DZ toxicity. We propose that NCUR exhibited better antioxidant effects and reduced the level of LPO due to its enhanced bioavailability.

DZ is reported to reduce the level of cellular antioxidant enzymes. Our results confirm that GSH contents significantly decreased in DZ-treated rats compared with that of control rats. However, treatment with NCUR efficiently restored the contents of GSH in the liver, kidney, and heart. Interestingly, in this study, GSH contents did not significantly decrease in the DZ+NCUR groups compared with those in the control group. This may be due to the direct free radical-scavenging activity of NCUR. Despite proof of its antioxidant activity, further studies are required to elucidate the underlying mechanisms.

Nanomicelles offer an alternative route of CUR delivery to the cells, thereby increasing its bioavailability and clinical efficacy. Results of this study confirm that the nanoparticulate formulation of CUR is effective in attenuating elevated ROS and LPO levels, and increasing GSH levels in the rat liver, heart, and kidney tissues; CUR as nanomicelles increased the levels of thiol-containing antioxidant enzymes to reduce cellular ROS levels (39). CUR is also reported to inhibit superoxide radical formation by increasing cellular GSH, which is the main non-protein thiol-containing antioxidant molecule present in living organisms (48, 49).

DZ easily disrupts the phospholipid-containing cell membranes due to its lipophilic nature. Therefore, it is worthwhile to explore whether the lipophilic and polyphenolic structure of NCUR is able to bind to and inactivate free-circulating DZ molecules. Hence, direct attachment of CUR and/or its metabolites to DZ may reduce cholinesterase enzymes inhibition by DZN, but it should be elucidated by further works. Furthermore, the high bioavailability of the synthesized NCUR (even single-dose) translates into increased intracellular concentrations of CUR, leading to enhanced antioxidant defense.

CUR prevents DZ-induced tissue damage by inhibiting the levels of lipoxygenases (50), free nitrogen radicals (51), hydroperoxides (52), and inflammatory molecules. Tetrahydrocurcumin, a major metabolite of CUR, also exerts potent antioxidant effects by scavenging free radicals and inhibiting lipid peroxidation (52). CUR exhibits antioxidant activities by increasing the levels of antioxidant enzymes, such as catalase, superoxide dismutase, and glutathione peroxidase. CUR acts as a Michael acceptor and reacts with thioredoxin and GSH to increase intracellular GSH. It is reported to be ten-fold more active than vitamin E. Methoxy and phenol groups in the phenyl ring along with a 1,3-diketone system contribute to the antioxidant activity (53).

A dose of 60 mg/kg of NCUR may be considered effective for its antioxidant effects. Therefore, CUR and NCUR reduce DZ toxicity due to their free radical-scavenging activities.

## Conclusion

The results obtained in this study suggested that both CUR and NCUR protect the liver, kidney, and heart from DZ-induced toxicity in rats. Administration of CUR and NCUR significantly decreased the serum biomarkers of oxidative damage and improved glutathione content in tissues. NCUR significantly attenuated DZ-induced oxidative damage in the heart, liver, and kidney compared to CUR. Additionally, it significantly inhibited the DZ-induced decrease in GSH levels in the heart, liver, and kidney compared to CUR. These results suggest that the synthesized NCUR outperforms CUR in attenuating oxidative tissue damage due to its high bioavailability. Therefore, we concluded that NCUR is superior to CUR in ameliorating the toxic effects of DZ in rats.

## References

[1] Shiri M, Navaei-Nigjeh M, Baeeri M, Rahimifard M, Mahboudi H, Shahverdi AR (2016). Blockage of both the extrinsic and intrinsic pathways of diazinon-induced apoptosis in PaTu cells by magnesium oxide and selenium nanoparticles. Int J Nanotechnol Nanomed.

[2] Aggarwal V, Deng X, Tuli A, Goh KS (2013). Diazinon—chemistry and environmental fate: A california perspective. Rev Environ Contam Toxicol.

[3] Abdou H, El Mazoudy R (2010). Oxidative damage, hyperlipidemia and histological alterations of cardiac and skeletal muscles induced by different doses of diazinon in female rats. J Hazard Mater.

[4] Boussabbeh M, Salem IB, Hamdi M, Fradj SB, Abid-Essefi S, Bacha H (2016). Diazinon, an organophosphate pesticide, induces oxidative stress and genotoxicity in cells deriving from large intestine. Environ Sci Pollut Res.

[5] Sargazi Z, Nikravesh MR, Jalali M, Sadeghnia HR, Rahimi Anbarkeh F, Mohammadzadeh L (2014). Diazinon-induced ovarian toxicity and protection by vitamins E. Iran J Toxicol.

[6] Soltaninejad K, Abdollahi M (2009). Current opinion on the science of organophosphate pesticides and toxic stress: a systematic review. Med Sci Monit.

[7] Aluigi M, Guida C, Falugi C (2010). Apoptosis as a specific biomarker of diazinon toxicity in NTera2-D1 cells. Chem Biol Interact.

[8] (2010). Shah MD, Iqbal M. Diazinon-induced oxidative stress and renal dysfunction in rats. Food Chem Toxicol.

[9] Jafari M, Salehi M, Ahmadi S, Asgari A, Abasnezhad M, Hajigholamali M (2012). The role of oxidative stress in diazinon-induced tissues toxicity in Wistar and Norway rats. Toxicol Mech Methods.

[10] Akturk O, Demirin H, Sutcu R, Yilmaz N, Koylu H, Altuntas I (2006). The effects of diazinon on lipid peroxidation and antioxidant enzymes in rat heart and ameliorating role of vitamin E and vitamin C. Cell Biol Toxicol.

[11] Krieger R (2001). Handbook of pesticide toxicology, two-volume set: principles and agents.

[12] Ammon HP, Wahl MA (1991). Pharmacology of Curcuma longa. Planta Medica.

[13] Calabrese V, Bates TE, Mancuso C, Cornelius C, Ventimiglia B, Cambria MT (2008). Curcumin and the cellular stress response in free radical-related diseases. Mol Nutr Food Res.

[14] Soltani B, Ghaemi N, Sadeghizadeh M, Najafi F (2016). Curcumin confers protection to irradiated THP-1 cells while its nanoformulation sensitizes these cells via apoptosis induction. Cell Biol Toxicol.

[15] Pandeya N (2005). Old wives’ tales: modern miracles—turmeric as traditional medicine in India. Trees Life J.

[16] He Y, Yue Y, Zheng X, Zhang K, Chen S, Du Z (2015). Curcumin, inflammation, and chronic diseases: how are they linked?. Molecules.

[17] Hatcher H, Planalp R, Cho J, Torti F, Torti S (2008). Curcumin: from ancient medicine to current clinical trials. Cell Mol Life Sci.

[18] Anand P, Kunnumakkara AB, Newman RA, Aggarwal BB (2007). Bioavailability of curcumin: problems and promises. Mol Pharm.

[19] Ghalandarlaki N, Alizadeh AM, Ashkani-Esfahani S (2014). Nanotechnology-applied curcumin for different diseases therapy. Biomed Res Int.

[20] Shaikh J, Ankola D, Beniwal V, Singh D, Kumar MR (2009). Nanoparticle encapsulation improves oral bioavailability of curcumin by at least 9-fold when compared to curcumin administered with piperine as absorption enhancer. Eur J Pharm Sci.

[21] Prasad S, Tyagi AK, Aggarwal BB (2014). Recent developments in delivery, bioavailability, absorption and metabolism of curcumin: the golden pigment from golden spice. Cancer Res Treat.

[22] Messarah M, Amamra W, Boumendjel A, Barkat L, Bouasla I, Abdennour C (2013). Ameliorating effects of curcumin and vitamin E on diazinon-induced oxidative damage in rat liver and erythrocytes. Toxicol Ind Health.

[23] Jaafari MR Formulation and preparation of nanomicelles containing curcuminoids for oral use. Iranian Patent Number 83515 2014.

[24] Rahimi HR, Mohammadpour AH, Dastani M, Jaafari MR, Abnous K, Mobarhan MG (2016). The effect of nano-curcumin on HbA1c, fasting blood glucose, and lipid profile in diabetic subjects: a randomized clinical trial. Avicenna J Phytomed.

[25] Akinyemi AJ, Onyebueke N, Faboya OA, Onikanni SA, Fadaka A, Olayide I (2017). Curcumin inhibits adenosine deaminase and arginase activities in cadmium-induced renal toxicity in rat kidney. J Food Drug Anal.

[26] Sak ME, Soydinc HE, Sak S, Evsen MS, Alabalik U, Akdemir F (2013). The protective effect of curcumin on ischemia-reperfusion injury in rat ovary. Int J Surg.

[27] Akinyemi AJ, Oboh G, Fadaka AO, Olatunji BP, Akomolafe S (2017). Curcumin administration suppress acetylcholinesterase gene expression in cadmium treated rats. Neurotoxicology.

[28] Katsumaro T, Tohru H (1985). Diazinon concentrations and blood cholinesterase activities in rats exposed to diazinon. Toxicol Lett.

[29] Izadi F, Jafari M, Bahdoran H, Asgari A, Divsalar A, Salehi M (2014). The role of N-acetyl cysteine on reduction of diazinon-induced oxidative stress in rat liver and kidney. J Rafsanjan Univ Med Sci.

[30] Fernandez F, Goudable C, Sie P, Ton-That H, Durand D, Suc J (1985). Low haematocrit and prolonged bleeding time in uraemic patients: effect of red cell transfusions. Br J Haematol.

[31] Moron MS, Depierre JW, Mannervik B (1979). Levels of glutathione, glutathione reductase and glutathione S-transferase activities in rat lung and liver. Biochim Biophys Acta Gen Subj.

[32] Barzegar A (2012). The role of electron-transfer and H-atom donation on the superb antioxidant activity and free radical reaction of curcumin. Food Chem.

[33] Hoeijmakers JH (2009). DNA damage, aging, and cancer. N Engl J Med.

[34] Tiwari H, Rao MV (2010). Curcumin supplementation protects from genotoxic effects of arsenic and fluoride. Food Chem Toxicol.

[35] Notarbartolo M, Poma P, Perri D, Dusonchet L, Cervello M, D’Alessandro N (2005). Antitumor effects of curcumin, alone or in combination with cisplatin or doxorubicin, on human hepatic cancer cells Analysis of their possible relationship to changes in NF-kB activation levels and in IAP gene expression. Cancer Lett.

[36] Yallapu MM, Jaggi M, Chauhan SC (2012). Curcumin nanoformulations: a future nanomedicine for cancer. Drug Discov Today.

[37] Yadav A, Lomash V, Samim M, Flora SJ (2012). Curcumin encapsulated in chitosan nanoparticles: a novel strategy for the treatment of arsenic toxicity. Chem Biol Interact.

[38] Srinivasan M, Prasad NR, Menon VP (2006). Protective effect of curcumin on γ-radiation induced DNA damage and lipid peroxidation in cultured human lymphocytes. Mutat Res Genet Toxicol Environ Mutagen..

[39] Sonkaew P, Sane A, Suppakul P (2012). Antioxidant activities of curcumin and ascorbyl dipalmitate nanoparticles and their activities after incorporation into cellulose-based packaging films. J Agric Food Chem.

[40] Alp H, Aytekin I, Esen H, Basarali K, Kul S (2011). Effects of caffeic acid phenethyl ester, ellagic acid, sulforaphane and curcumin on diazinon induced damage to the lungs, liver and kidneys in an acute toxicity rat model. Kafkas Univ Vet Fak Derg.

[41] El-Shenawy NS, El-Salmy F, Al-Eisa RA, El-Ahmary B (2010). Amelioratory effect of vitamin E on organophosphorus insecticide diazinon-induced oxidative stress in mice liver. Pestic Biochem Physiol.

[42] Ozer J, Ratner M, Shaw M, Bailey W, Schomaker S (2008). The current state of serum biomarkers of hepatotoxicity. Toxicol.

[43] Oruç EÖ, Usta D (2007). Evaluation of oxidative stress responses and neurotoxicity potential of diazinon in different tissues of Cyprinus carpio. Environ Toxicol Pharmacol.

[44] Isik I, Celik I (2008). Acute effects of methyl parathion and diazinon as inducers for oxidative stress on certain biomarkers in various tissues of rainbowtrout (Oncorhynchus mykiss). Pestic Biochem Physiol.

[45] Priscilla DH, Prince PSM (2009). Cardioprotective effect of gallic acid on cardiac troponin-T, cardiac marker enzymes, lipid peroxidation products and antioxidants in experimentally induced myocardial infarction in Wistar rats. Chem Biol Interact.

[46] Hariri AT, Moallem SA, Mahmoudi M, Memar B, Hosseinzadeh H (2010). Sub-acute effects of diazinon on biochemical indices and specific biomarkers in rats: protective effects of crocin and safranal. Food Chem Toxicol.

[47] Kose A, Gunay N, Yildirim C, Tarakcioglu M, Sari I, Demiryurek AT (2009). Cardiac damage in acute organophosphate poisoning in rats: Effects of atropine and pralidoxime. Am J Emerg Med.

[48] Tirkey N, Kaur G, Vij G, Chopra K (2005). Curcumin, a diferuloylmethane, attenuates cyclosporine-induced renal dysfunction and oxidative stress in rat kidneys. BMC Pharmacol.

[49] Singh RP, Sharad S, Kapur S (2004). Free radicals and oxidative stress in neurodegenerative diseases: relevance of dietary antioxidants. J Indian Acad Clin Med.

[50] Kumar A, Dogra S, Prakash A (2009). Protective effect of curcumin (Curcuma longa), against aluminum toxicity: Possible behavioral and biochemical alterations in rats. Behav Brain Res.

[51] Rao M (1997). Nitric oxide scavenging by curcuminoids. J Pharm Pharmacol.

[52] Somparn P, Phisalaphong C, Nakornchai S, Unchern S, Morales NP (2007). Comparative antioxidant activities of curcumin and its dimethoxy and hydrogenated derivatives. Biol Pharm Bull.

[53] Ramawat KG, Dass S, Mathur M (2009). Herbal drugs: ethnomedicine to modern medicine.

